# Effect of Surface Polishing on Physical Properties of an Occlusal Splint Material for Additive Manufacturing under Protection Gas Post-Curing Condition

**DOI:** 10.3390/polym15030625

**Published:** 2023-01-25

**Authors:** Junichiro Wada, Kanae Wada, Mona Gibreel, Noriyuki Wakabayashi, Tsutomu Iwamoto, Pekka K. Vallittu, Lippo Lassila

**Affiliations:** 1 Department of Biomaterials Science, Turku Clinical Biomaterials Centre–TCBC, Institute of Dentistry, University of Turku, Itäinen Pitkäkatu 4B, 20520 Turku, Finland; 2 Department of Advanced Prosthodontics, Tokyo Medical and Dental University–TMDU, 1-5-45 Yushima, Bunkyo-ku, Tokyo 113–8510, Japan; 3 Department of Pediatric Dentistry/Special Needs Dentistry, Tokyo Medical and Dental University–TMDU, 1-5-45 Yushima, Bunkyo-ku, Tokyo 113–8510, Japan; 4 Wellbeing Services County of South-West Finland, Lemminkäisenkatu 23, 20520 Turku, Finland

**Keywords:** additive manufacturing, degree of carbon double bond conversion, nitrogen gas, occlusal splint, physical property, post-curing, 3D printer

## Abstract

The aim of this study was to evaluate the effect of surface polishing as well as the post-curing atmospheres (air and nitrogen gas) on the physical properties of an occlusal splint material for additive manufacturing. Flexural strength, flexural modulus, Vickers hardness number (VHN), degree of carbon double bond conversion (DC), water sorption (W_SP_), and water solubility (W_SL_) were evaluated. Surface polishing significantly affected the evaluated properties. Regardless of the post-curing atmosphere, flexural strength, flexural modulus, VHN, and DC showed significantly higher values for the polished specimens when compared with the unpolished ones, while W_SP_ and W_SL_ were significantly lower for the polished specimens. Unpolished specimens post-cured at nitrogen gas showed significantly higher VHN and DC values. However, the effect of the post-curing at a nitrogen gas atmosphere was non-significant in polished specimens. The current results suggested that surface polishing plays a role in the physical properties of the evaluated occlusal splint material and can enhance all the evaluated properties regardless of the post-curing atmosphere. Meanwhile, the post-curing at a nitrogen gas atmosphere can enhance the VHN and DC but its effect is confined only to the surface layers, which can be removed during surface polishing.

## 1. Introduction

**Figure 1:**
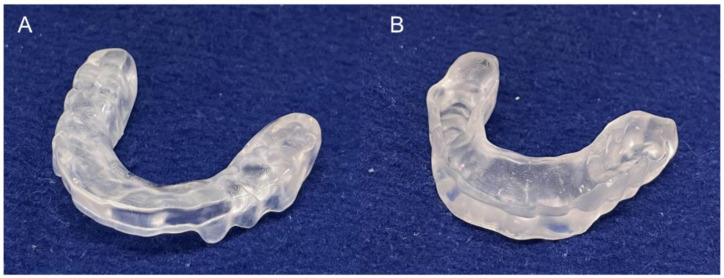
3D-printed occlusal splint. (**A**): occlusal surface on which adjustment including polishing is frequently required, and (**B**): fitting surface on which adjustment is hardly necessary.

The fabrication of prostheses and oral appliances using digital technology has been recently applied in several clinical situations [1,2,3]. The mainstream methods for the fabrication of dental objects using digital technologies can be classified into milling and additive manufacturing, which is well known as 3D printing [4,5,6]. As reported in previous studies, objects fabricated by milling have advantages in terms of strength and fitting accuracy [7,8], while additive manufacturing can provide objects with minimal cutting material waste at a low cost [9]. Generally, except for interim prostheses, milling manufacturing is recommended for the fabrication of fixed crowns and bridges that need enough strength and accurate fitting [10]. Nevertheless, additive manufacturing is suitable for the fabrication of occlusal splints (OS) that are expected to be clinically used for a short time and do not demand higher aesthetics (Figure 1) [11].

Additive manufacturing systems can be mainly classified into inkjet printing, extrusion printing, selective laser melting, and stereolithography printing [12,13,14,15]. In this classification, the stereolithography printing system has been widely used for OS fabrication. For the stereolithography printing system, digital light processing (DLP) and liquid crystal display (LCD) are mainly used as the principles of pattern formation for 3D printers [16]. Thanks to the increased light intensity, DLP printers can produce items more effectively, leading to the widespread development of dental materials for DLP-based stereolithography printing systems [17,18]. On the other hand, previous studies reported that LCD printers could provide objects with a smoother surface and a higher degree of conversion than DLP printers [19,20]. Although LCD printers might have several advantages, only a few studies have been performed on objects printed using LCD printers.

The post-curing procedure is considered as an essential step after stereolithographic printing. Previous studies reported that post-curing could enhance the degree of surface conversion [21] and improve several physical properties of 3D-printed objects [22]. Nevertheless, some other studies found that the effect of post-curing conditions was limited only to the surface layers but not the core [19,20]. Adjustment is hardly necessary for the fitting surface of OS fabricated by 3D printers, suggesting that the post-curing conditions would have an impact on the inner surface. However, adjustments on the occlusal surface of OS in the form of cutting and polishing is frequently required to achieve appropriate occlusal contact with the antagonists, resulting in the removal of surface layers from the occlusal surface of OS. Nevertheless, there is no report evaluating the effect of post-curing methods and surface polishing on the physical properties of 3D-printed occlusal splints.

Therefore, the aim of this study was to evaluate the effect of surface polishing on the physical properties of 3D-printed OS materials post-cured at different atmospheres. The evaluated properties were flexural strength, flexural modulus, surface microhardness, degree of carbon double bond conversion, water sorption, and water solubility. The null hypotheses were as follows: (1) the surface polishing would have no impact on the evaluated properties of 3D-printed OS materials, and (2) the post-curing method would have no impact on the evaluated properties.

## 2. Materials and Methods

### 2.1. Specimen Fabrication

**Figure 2:**
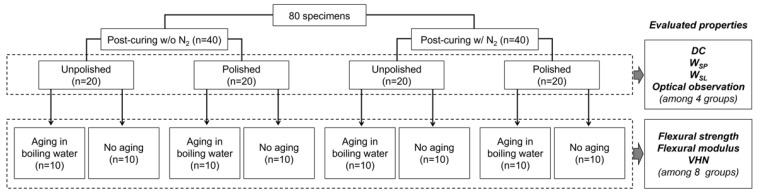
Flowchart of specimen fabrication. N_2_: nitrogen gas; DC: degree of carbon double bond conversion; W_SP_: water sorption; W_SL_: water solubility; and VHN: Vickers hardness number. Post-curing w/o N_2_: stroboscopic post-curing in the air atmosphere. Post-curing w/ N_2_: stroboscopic post-curing at a nitrogen gas atmosphere.

**Figure 3:**
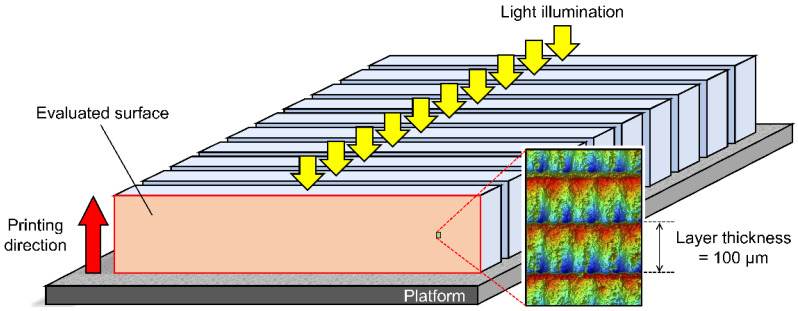
The printing direction (red arrow), direction of light illumination (yellow arrows), and evaluated surface for Vickers hardness number (VHN) testing and optical observation.

Figure 2 summarizes the preparation procedures and classification of specimens. Eighty bar-shaped specimens were made of a photopolymerizing material used for additive-manufactured OS (KeySplint^®^ Hard, Keystone Industries GmbH, Singen, Germany) which is based on methacrylate chemistries. All specimens were additive-manufactured at room temperature (23 ± 1 °C) using an LCD printer of which the LED wavelength was 405 nm (Creo™ C5, PLANMECA OY, Helsinki, Finland) with a print orientation of 90° and a layer thickness of 100 µm (Figure 3), followed by rinsing with isopropanol for 10 min in an ultrasonic cleaning unit (Quantrex^®^ 90, L&R Ultrasonics, Kearny, NJ, USA). Afterward, half of the specimens (n = 40) underwent stroboscopic post-curing with 2000 flashes on each surface (Otoflash G171, BEGO GmbH & Co, Bremen, Germany) in the air with an absence of nitrogen gas (N_2_) atmosphere (without N_2_), while the other half (n = 40) underwent stroboscopic post-curing at an N_2_ atmosphere (with N_2_). Each post-cured group was divided into the following two subgroups based on the polishing condition: unpolished and polished subgroups (n = 20/subgroup). For the polished subgroups, wet polishing was performed using a silicon carbide grinding paper 4000-grit with a grain size of 5 µm (SiC Paper #4000, Struers LLC, Cleveland, USA). The size of each specimen was standardized with a dimension of 3.0 mm × 10.0 mm × 60.0 mm (±0.2 mm). In addition, half of the specimens in each subgroup (n = 10) were exposed to accelerated aging procedures by boiling in distilled water for 16 h as the aging procedure (aged specimens), while the other half (n = 10) were not boiled (non-aged specimens).

### 2.2. Flexural Strength and Modulus Testing

For each specimen, a 3-point bending test was performed to assess the flexural strength (MPa) and elastic modulus (GPa) in the air atmosphere at room temperature by a universal testing machine (Model LRX; Lloyds Instruments Ltd., Hampshire, UK) using a load cell with a capacity of 2500 N. The crosshead speed was 5.0 mm/min and the span length was 50 mm. Each test was detected to be finished based on one of the following test conditions: 1) the reduction in the load reached 10% of the maximum load, and 2) the deflection of the specimen reached 12 mm. Additionally, the number of specimens broken during the testing was counted and the broken ratio (%) was calculated.

### 2.3. Surface Microhardness (Vickers Hardness)

For each subgroup, surface microhardness was measured at 10 different regions on two randomly selected specimens using a Vickers hardness testing device (Duramin-5, Struers, Ballerup, Denmark). The diagonals of the square-based pyramid indentation impressed on the specimen were measured, and the Vickers hardness number (VHN) was calculated using the following equation:

(1) $$\mathrm{VHN}=\frac{0.1891\times F}{d^{2}}$$

where F is the indenting force (N) and d is the mean length of two diagonals of the pyramid indentation (mm).

### 2.4. Degree of Carbon Double Bond Conversion (DC)

The degree of carbon double bond conversion (DC) on the surface of five specimens randomly selected from each non-aged group was measured using a Fourier transform infrared (FTIR) spectrometer (Frontier FT–IR spectrometer, PerkinElmer, Llantrisant, UK) based on the unpolymerized material as the control. The absorbance intensity of the aliphatic C=C absorbance peak ratio at 1638 cm^−1^ against the reference aromatic peak at 1600 cm^−1^ was measured for both unpolymerized material and selected specimens. The values of DC were calculated as a percentage (%) using the following equation:

(2) $$\mathrm{DC}=\left(1-\frac{C_{1638}/C_{1600}}{U_{1638}/U_{1600}}\right)\times 100$$

where C_1638_ is the polymerized aliphatic peaks ratio; C_1600_ is the polymerized aromatic peaks ratio; U_1638_ is the unpolymerized aliphatic peaks ratio; and U_1600_ is the unpolymerized aromatic peaks ratio.

### 2.5. Water Sorption and Solubility

Based on the previous studies, water sorption (W_SP_) and solubility (W_SL_) were measured using eight non-aged specimens randomly selected from each group [19,20,23,24]. During the first drying cycle procedures, the specimens were dried in a vacuum desiccator containing freshly dried silica at 37 ± 1 °C for 22 h, and then stored at 23 ± 1 °C for 2 h. The initial mass (m_1_) of each specimen was measured by a digital analytical balance (XS105; Mettler Toledo, Greifensee, Switzerland). Then, the drying procedure was repeated until the decrease in mass during a single day was not more than 0.1 mg for each specimen. After finishing the first drying procedure, the specimens were immersed in 40.0 mL of distilled water and stored at 37 ± 1 °C for 30 days. At 1, 2, 3, 7, 14, 21, 28, and 30 days after immersion into the water, the specimens were removed from the water and carefully dried with absorbent paper for 60 s. Then, the mass of each specimen was measured. The mass at 30 days after the first water immersion was recorded and considered as m_2_. Similarly as the first drying procedure, specimens were dried during the second drying cycle until a consistent mass (m_3_) of each specimen was achieved. Finally, W_SP_ and W_SL_ were estimated in percentages using the following equations:

(3) $$W_{\mathrm{SP}}=\left(\frac{m_{2}-m_{3}}{m_{1}}\right)\times 100$$

and

(4) $$W_{\mathrm{SL}}=\left(\frac{m_{1}-m_{3}}{m_{1}}\right)\times 100$$

where m_1_ is the initial mass of the specimen after the first drying cycle (mg); m_2_ is the mass of the specimen at 30 days after water immersion (mg); and m_3_ is the constant mass of the specimen achieved after the second drying cycle (mg).

### 2.6. Optical Observation of Surface Condition

One specimen was randomly selected from each non-aged group for optical observation of the surface condition using a 3D optical profilometer (OP) (ContourGT–I, Bruker Nano, Inc., Tucson, AZ, USA). The size of the regions of interest for observation was 236.0 µm × 314.2 µm.

### 2.7. Statistical Analysis

Since the equality of variance was detected by the Levene test in each acquired data, parametric testing methods were used. Three predictors including the post-curing atmosphere, polishing, and aging were tested for the flexural strength, flexural modulus, and VHN using a 3-way analysis of variance (ANOVA), while the two predictors including the post-curing atmosphere and polishing were tested for the DC, W_SP_, and W_SL_ using a 2-way ANOVA. Additionally, all the acquired data were statistically compared among the groups using a 1-way ANOVA and Tukey multiple comparisons as a *post hoc* analysis. Regarding the broken ratio of specimens during the flexural strength and modulus test, the Chi-squared test was performed for the comparison between unpolished and polished groups independently within each subgroup and classified based on the post-curing atmosphere and aging procedure. Statistical software (IBM SPSS Statistics v28.0, IBM, Redmond, WA, USA) was used for all the statical analyses with the significance level set at 0.05.

## 3. Results

The data of unpolished subgroups overlapped with the data in the previous study [19]. Table 1 shows the *p*-values acquired by multiple ANOVA. The 3-way ANOVA revealed that the post-curing atmosphere, polishing, and aging significantly affected the flexural strength, flexural modulus, and VHN (*p* < 0.001). Additionally, the 2-way ANOVA revealed that the polishing significantly affected the DC, W_SP_, and W_SL_ (*p* < 0.001), while the post-curing atmosphere significantly affected the DC (*p* < 0.001) and W_SP_ (*p* = 0.038). On the other hand, no significant effect was found for the post-curing atmosphere on the W_SL_ (*p* = 0.607).

**Figure 4:**
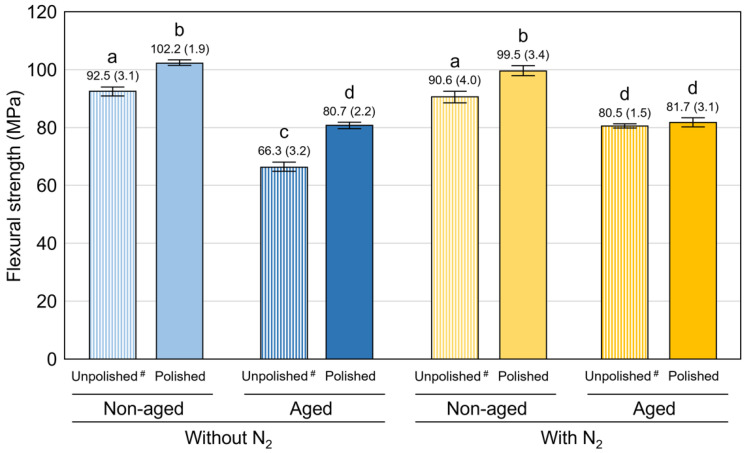
Mean values (standard deviations) of flexural strength. The same superscripted letters indicate groups not statistically significantly different when compared by 1-way ANOVA and post hoc analysis with Tukey multiple comparisons. ^#^: data of unpolished subgroups were quoted from the previous study [19].

**Figure 5:**
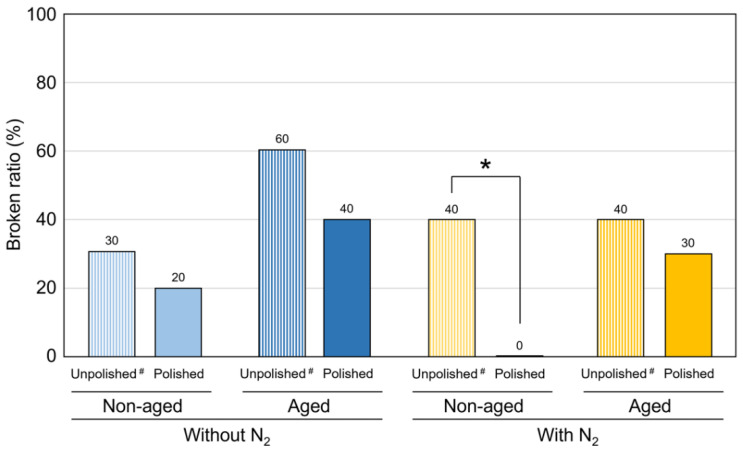
The broken ratio of specimens broken during the flexural strength and modulus test. The asterisk (^*^) indicates a statistically significant difference between the unpolished and polished groups when compared by the Chi-squared test. It was noted that the polished group showed a lower broken ratio than the unpolished group in each subgroup. ^#^: data of the unpolished subgroups were quoted from the previous study [19].

Figure 4 shows the mean values and standard deviations of the flexural strength, and Table 2 shows the mean values and standard deviations of the flexural modulus and VHN. Polishing generally resulted in a statistically significant improvement of the evaluated physical properties except for the flexural strength and modulus in the aged group post-cued with N_2_ (*p* < 0.001 for all). Only in unpolished subgroups, the post-curing at N_2_ significantly enhanced the flexural strength and modulus after the aging in boiling water, while it significantly enhanced the VHN both in non-aged (*p* < 0.001 for all) and aged specimens (*p* < 0.001 for all). Additionally, aging in boiling water significantly deteriorated the evaluated physical properties (*p* < 0.05), with the exception of the flexural modulus (*p* = 1.000) and VHN (*p* = 0.091) of the unpolished specimens post-cured with N_2_. Figure 5 shows the broken ratio of specimens broken during the flexural strength and modulus test. Polishing generally resulted in a lower broken ratio in all subgroups. The polished specimens were never broken, and the polished group revealed a significantly lower broken ratio than the unpolished group in the non-aged subgroup post-cured with N_2_ (*p* = 0.025).

**Figure 6:**
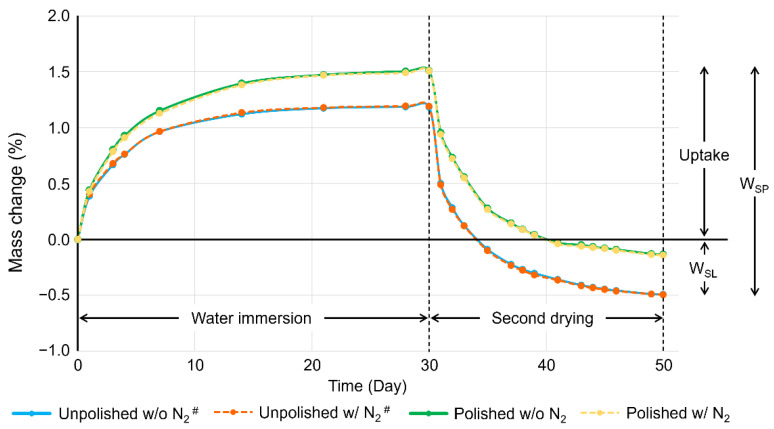
The representative plots of mass changes (%) against time during the water immersion and the second drying procedures. w/o N_2_: stroboscopic post-curing in the air atmosphere. Post-curing w/ N_2_: stroboscopic post-curing at a nitrogen gas atmosphere. It was noted that saturation was achieved 14 days after water immersion and drying was completed 20 days after drying began in all groups. ^#^: data of unpolished subgroups were quoted from the previous study [19].

**Figure 7:**
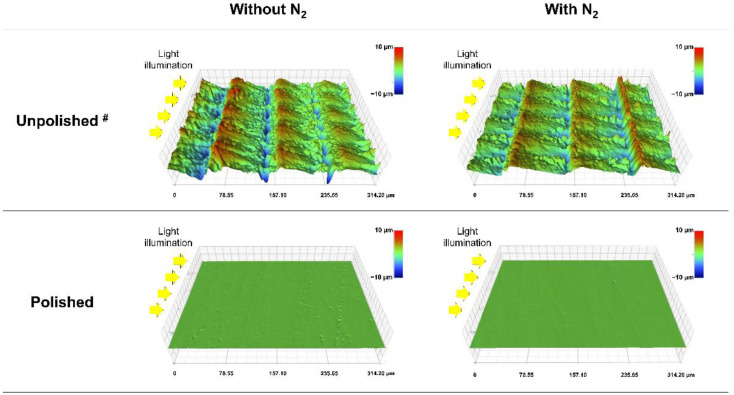
Optical profilometer (OP) images in typical specimens. The yellow arrows represent the light illuminating direction. It was noted that the wavelike structures with a height of less than 10 µm were observed only in polished specimens and there was no difference between specimens post-cured without and with N_2_. ^#^: images of unpolished subgroups were quoted from the previous study [19].

Table 3 shows the mean values and standard deviations of the DC, W_SP_, and W_SL_. Comparing the DC between the unpolished subgroups, the one post-cured without N_2_ revealed significantly lower DC than that post-cured with N_2_ (*p* < 0.001), while there was no significant difference among polished subgroups post-cured without and with N_2_. The W_SP_ and W_SL_ of the polished subgroups were significantly lower than those of the unpolished subgroups (*p* < 0.05), while the post-curing atmosphere did not significantly affect W_SP_ and W_SL_ in both unpolished and polished subgroups. Figure 6 shows the mass changes (%) of each group against time during the water immersion and second drying procedures. Saturation was achieved 14 days after water immersion. Nevertheless, the drying was completed 20 days after drying began in all groups. Figure 7 shows the OP images in typical specimens. The wavelike structures with a height of less than 10 µm were observed only in polished specimens, and there was no optical difference between specimens post-cured without and with N_2_.

## 4. Discussion

This study demonstrated the effect of post-curing atmospheres and surface polishing on the physical properties, including flexural strength, flexural modulus, surface microhardness (VHN), DC, W_SP_, and W_SL_ in an additive-manufactured OS material fabricated by an LCD printer. The overall results rejected the two null hypotheses and revealed that the stroboscopic post-curing at an N_2_ atmosphere and surface polishing enhanced some of the evaluated properties of the additive-manufactured OS material.

Under the air atmosphere, an oxygen-inhibited layer could be formed on the surface of polymerized objects [25]. In this study, the post-curing with N_2_ enhanced the evaluated properties except for the W_SP_ and W_SL_. This was in agreement with the previous studies, where the nitrogen post-curing atmosphere could prevent the oxygen-inhibited layer formation [21,26]. On the other hand, some of the evaluated physical properties were significantly improved by a surface polishing, which might be due to the removal of the surface layer of the specimens. In addition, as shown in Figure 7, polishing results in more flat and smooth surfaces for the specimens and eliminates irregularities, including grooves that might act as a pre-crack. Therefore, the geometrical flattening of the surface might be crucial for the flexural strength and modulus.

The post-curing with N_2_ significantly enhanced the VHN and DC of the non-aged unpolished subgroups, while it had no impact on the physical properties of aged and non-aged polished subgroups. This finding indicates that the effect of post-curing atmospheres would be only restricted to the surface layers which are usually removed during surface polishing, coinciding with the findings of previous studies [19,20]. Furthermore, there was no significant difference in flexural strength and modulus between aged unpolished and polished subgroups post-cured with N_2_, suggesting that the enhancement of those properties gained by the post-curing with N_2_ against the aging would be comparable to that gained by surface polishing. It was also suggested that the post-curing with N_2_ could diminish the deterioration of flexural strength and modulus during aging, while it would have no impact on those properties in the additive-manufactured OS before aging. Additionally, surface polishing had no significant impact on DC in groups post-cured with N_2_.

Unlike fixed prostheses where the inner surfaces never appear outside, OS is an oral appliance that includes the outer (occlusal) surface which requires adjustments with surface layer removal, and the inner (fitting) surface which hardly needs any adjustment. The occlusal surface of the OS should resist mechanical stress from the antagonist. In this study, the surface layers with a thickness of around 250 µm were removed from the surface of each specimen during the polishing procedures. Polishing seemed to have a significant role in enhancing flexural strength, flexural modulus, and VHN regardless of the post-curing atmosphere. This finding highlights the clinical significance of occlusal surface adjustment procedures, including cutting and polishing for the OS.

Materials with a higher DC could present lower levels of cytotoxicity [27,28], supposing that the DC is a clinically critical factor for the inner surfaces of the OS. The DC of unpolished subgroups was dramatically improved by the post-curing with N_2_, suggesting that N_2_ used as the post-curing atmosphere would be clinically recommended, especially from the viewpoint of biological compatibility. Meanwhile, W_SP_ and W_SL_ were hardly enhanced by the post-curing with N_2_. W_SP_ and W_SL_ are related to the dimensional change in the OS leading to internal stresses that adversely impact its longevity [23,29], indicating that it is clinically important to maintain W_SP_ and W_SL_ at a lower level to prevent the fracture of the OS and gain its long-term success. Therefore, even for OSs with an inner surface that needs almost no adjustment, it is necessary to investigate different methods to decrease W_SP_ and W_SL_ in further studies.

Only one printer with an LCD system was used in this study. Furthermore, the layer thickness during printing was unified to 100 µm. The printing layer thickness of 3D-printed OS materials can have an impact on their physical properties [30]. Future studies should be considered to determine the effect of surface polishing and/or post-curing atmospheres on additive-manufactured OSs with various layer thicknesses. Finally, the aging condition used in this study was not identical to the actual clinical scenario. However, the accelerated aging procedure has been reported to be efficient in simulating hydrolytic deteriorations and thermal breakdown [31,32].

## 5. Conclusions

Within the limitations of this study, it can be concluded that surface polishing can enhance flexural strength, flexural modulus, surface microhardness, degree of carbon double bond conversion, water sorption, and water solubility of the evaluated additively manufactured occlusal splint material regardless of the post-curing atmosphere. The post-curing atmosphere also plays a role in the physical properties of the evaluated material. Stroboscopic post-curing at a nitrogen gas atmosphere can enhance surface microhardness and the degree of double carbon bond conversion of the evaluated splint material and minimize the aging deterioration of the flexural strength and modulus. However, the effect of post-curing atmospheres is limited only to the surface layers which can be removed by surface polishing.

## Figures and Tables

**Table 1:** *p*-values acquired by 3- and 2-way ANOVA for the evaluated mechanical properties.

Predictor □	Flexural Strength ^#^	Flexural Modulus ^#^	VHN ^#^	DC ^$^	W_SP_^$^	W_SL_^$^
post-curing atmosphere	<0.001 ^*^	<0.001 ^*^	<0.001 ^*^	<0.001 ^*^	0.038 ^*^	0.607
polishing	<0.001 ^*^	<0.001 ^*^	<0.001 ^*^	<0.001 ^*^	<0.001 ^*^	<0.001 ^*^
aging	<0.001 ^*^	<0.001 ^*^	<0.001 ^*^	–	–	–

^#^: *p*-values acquired by 3-way ANOVA; ^$^: *p*-values acquired by 2-way ANOVA; and ^*^: statistically significant.

**Table 2:** Mean values and standard deviations of flexural modulus and VHN, and results of 1-way ANOVA statistical analysis.

Post-Curing Atmosphere	Aging	Polishing	Flexural Modulus (GPa)		VHN	
□	□	□	□	^$^	□	^$^
Without N_2_	non-aged	Unpolished ^#^	2.21 (0.11)	a	12.5 (0.4)	a
□	□	Polished	2.60 (0.11)	b	16.9 (0.2)	b
□	aged	Unpolished ^#^	1.70 (0.14)	c	12.1 (0.4)	c
□	□	Polished	2.13 (0.11)	a	12.7 (0.5)	a
□	□	□	□	□	□	□
With N_2_	non-aged	Unpolished ^#^	2.13 (0.13)	a	15.7 (0.5)	d
□	□	Polished	2.54 (0.12)	b	17.0 (0.2)	b
□	aged	Unpolished ^#^	2.11 (0.07)	a	15.3 (0.5)	d
□	□	Polished	2.12 (0.13)	a	12.5 (0.4)	a

^#^: data of unpolished subgroups were quoted from the previous study [19], and ^$^: the same superscripted letters indicate groups not statistically significantly different when compared by 1-way ANOVA and post hoc analysis with Tukey multiple comparisons.

**Table 3:** Mean values and standard deviations of DC, W_SP_, and W_SL_, and results of 1-way ANOVA statistical analysis.

Post-Curing Atmosphere	Polishing	DC (%)		W_SP_ (%)		W_SL_ (%)	
			^$^		^$^		^$^
Without N_2_	Unpolished ^#^	64.7 (6.4)	a	1.685 (0.004)	a	0.495 (0.020)	a
–	Polished	96.0 (3.0)	b	1.657 (0.006)	b	0.139 (0.022)	b
–	□	□	□	□	□	□	□
With N_2_	Unpolished ^#^	92.3 (4.5)	b	1.696 (0.006)	a	0.496 (0.036)	a
–	Polished	97.3 (3.7)	b	1.664 (0.017)	b	0.138 (0.022)	b

^#^: data of unpolished subgroups were quoted from the previous study [19], and ^$^: the same superscripted letters indicate groups not statistically significantly different when compared by 1-way ANOVA and post hoc analysis with Tukey multiple comparisons.

## Data Availability

The data presented in this study are available on reasonable request from the corresponding author.
